# From Perceived Stress to Demoralization in Parkinson Disease: A Path Analysis

**DOI:** 10.3389/fpsyt.2022.876445

**Published:** 2022-05-10

**Authors:** John M. de Figueiredo, Boheng Zhu, Amar Patel, Robert Kohn, Brian B. Koo, Elan D. Louis

**Affiliations:** ^1^Department of Psychiatry, Yale University School of Medicine, New Haven, CT, United States; ^2^Department of Psychological Medicine, Peking Union Medical College Hospital, Chinese Academy of Medical Sciences and Peking Union Medical College, Beijing, China; ^3^Department of Neurology, Yale University School of Medicine, New Haven, CT, United States; ^4^Department of Psychiatry, Brown University School of Medicine, Providence, RI, United States; ^5^Department of Neurology and Neurotherapeutics, University of Texas Southwestern, Dallas, TX, United States

**Keywords:** demoralization, subjective incompetence, depression, anxiety, stress, Parkinson's disease, suicide/suicidal ideation/suicidal behavior

## Abstract

**Objectives:**

The objective of this study was to determine whether depression and anxiety are mediators between perceived stress and demoralization via a loss of the cognitive map to get out of the predicament manifesting as subjective incompetence.

**Methods:**

Ninety-five consecutive outpatients with Parkinson's disease were evaluated for perceived stress, depression, anxiety, subjective incompetence, and demoralization using reliable and valid scales. Inclusion criteria were ages 40–90, intact cognition, and no current history of substance use. The setting was a Movement Disorders Clinic at a university-affiliated hospital. The outcome variable was demoralization, selected *a priori*. Mediators between perceived stress and demoralization were examined using path analysis.

**Results:**

Depression, anxiety, and subjective incompetence were mediators between perceived stress and demoralization. Among all variables, subjective incompetence was the largest contributor to demoralization. Depression connected to demoralization indirectly via subjective incompetence (β = 0.25, *p* < 0.001), whereas anxiety bypassed subjective incompetence (β = −0.01, *p* = 0.882), connecting directly to demoralization (β = 0.37, *p* = 0.008).

**Conclusion:**

Early treatment and reversal of subjective incompetence and anxiety could potentially prevent the escalation of demoralization and the associated disruption in health-related quality of life and eventual suicide.

## Introduction

Parkinson's disease is the second most common neurodegenerative disease in the United States and the fastest growing neurological disease (1, 2). Although classified as a “movement disorder,” Parkinson's disease frequently displays non-motor symptoms, including depression and anxiety, sometimes as prodromal manifestations, often associated with faster decline of motor and cognitive functions (3). Only recently, however, has attention been given to whether Parkinson's disease patients diagnosed as “depressed” or “anxious” might also be demoralized.

By generalizing and modifying slightly a definition proposed for cancer patients, distress may be defined as follows: “An unpleasant emotional experience of a psychological, social, and/or spiritual nature that may interfere with the ability to cope effectively with a stressful situation. Distress extends along a spectrum, ranging from common non-pathological feelings of vulnerability, sadness, and fears to problems that can become disabling, such as depression, anxiety, panic, social isolation, and spiritual crisis” (4, 5). Distress should not be confused with demoralization. Although several definitions of demoralization have been proposed, consensus has been emerging that demoralization is manifested by expressions of distress, such as loss of meaning and purpose, disheartenment, dysphoria, sense of failure, as well as inability to cope and feelings of irrelevance, futility, existential despair, or combinations thereof (6, 7). Subjective incompetence is the expression of the absence or loss of a cognitive map to get out of the predicament, a self-perceived incapacity to deal effectively with a stressful situation. Subjective incompetence has been called the “clinical hallmark of demoralization”, meaning that the presence of subjective incompetence in the context of distress signals the occurrence of demoralization (8, 9). Subjective incompetence may or may not occur in the context of distress. When it occurs in the context of distress, subjective incompetence is the clinical hallmark of demoralization, i.e., the presence of subjective incompetence signals the occurrence of demoralization (8, 9). Central to demoralization is the feeling of loss of hope. Hope has been defined as “the process of thinking about one's goals along with the motivation to move toward those goals (agency), and the ways to achieve those goals (pathways)” (10, 11). Helplessness is the belief and feeling that the person himself/herself has no power to improve his/her stressful situation. Hopelessness is the belief and feeling that nothing can be done by anyone to make the stressful situation any better. It has been proposed that subjective incompetence may progress to helplessness and hopelessness, this progression constituting what may be called the “demoralization cascade”, i. e., some people who have subjective incompetence become helpless; some who believe and feel they are helpless become hopeless; and some who believe and feel they are hopeless become suicidal (12). As Abramson et al. noted, hopelessness always involves helplessness, i.e., hopelessness is a subset of helplessness, and, therefore, when hopelessness occurs, helplessness also occurs (13). This conceptualization of “demoralization cascade” is consistent with research. For example, hopelessness has been shown to be distinct from depression and a stronger predictor of suicide than depression among psychiatric patients (14–20). As Constanza et al. asserted, hopelessness and suicidal ideation play a primary role in the suffering of patients with Parkinson's disease and other neurological diseases and are better explained as a form of existential despair than as a manifestation of a depressive disorder (21).

Demoralization is a transdiagnostic categorization, i.e., a condition that may occur in patients with a variety of psychiatric and non-psychiatric medical diagnoses. Demoralization occurs in one-third of patients or more in medical settings, with a prevalence of 20–30% in community settings (22, 23). Depression and demoralization may co-occur, but they have different trajectories and require different interventions. In demoralization, there is no anhedonia or anergia and there is a willingness to take action, but the course of action (“pathways”) is uncertain. In depression, willpower (“agency”) is decreased or lacking (abulia) even when the course of action (“pathways”) is known. The uncertainty about the course of action characteristic of demoralization is a manifestation of subjective incompetence (24).

Research by our team found that in Parkinson's disease, demoralization is highly associated with depression, but not completely; lifetime histories of both depression and demoralization are more likely in patients than in controls; demoralization explains disruptions in health-related quality of life better than depression; and demoralized patients are more likely than controls to have suicidal ideation (25–27). Here we report a path analysis to determine whether depression, anxiety, and subjective incompetence may be viewed as mediators between perceived stress and demoralization. These variables were selected because previous research found that demoralization in patients with progressive disease is consistently associated with poorly treated depression or anxiety (28) and subjective incompetence has been shown to be a clinical hallmark of demoralization in cancer patients (29, 30). Within the path analysis framework, another aim of this research was to determine whether depression and anxiety influence and are followed by demoralization via subjective incompetence and to describe the reciprocal relationships of the mediators. Knowing these mediators is essential because controlling them would interrupt the demoralization cascade and potentially prevent disruptions in health-related quality of life, meaninglessness, existential despair, and eventual suicide.

## Methods

Consecutive outpatients with Parkinson's disease were recruited from the Movement Disorders Clinic at Yale-New Haven Hospital. Parkinson's disease was diagnosed by a movement-disorders neurologist (A.P.) using the UK Brain Bank Society criteria (31).

### Participants

Inclusion criteria were age of 40 to 90 years and English comprehension/literacy. Exclusion criteria were substance abuse, history of dementia, and terminal illness. A total of 133 eligible patients were invited to participate. Of these, 38 declined (not interested). Those who declined were similar to the participants in terms of age and sex distributions but were more likely to be in Hoehn and Yahr stages III or IV (32).

### Data Sources and Assessments

Questionnaires were administered in person by trained research assistants after clinic appointments. Participants reported their age, sex, race/ethnicity, marital status, education, household size, cigarette smoking, and drinking habits. A chart review validated the self-reported information about years since Parkinson's disease diagnosis and treatment with deep brain stimulation, antiparkinsonian medications, and levodopa. Other diseases or disorders were reported with a standard systems-review form.

Participants were classified according to the Hoehn and Yahr stage (32), assessed for dyskinesia, and evaluated for motor function using the revised Movement Disorders Society Sponsored Unified Parkinson's disease Rating Scale, Part III (MDS-UPDRS-m) (33). The scoring of Hoehn and Yahr scale is based on the history given by the patients and their caregivers and a medical examination and distinguishes five stages: (1). Unilateral involvement usually with minimal or no functional disability; (2). Bilateral or midline involvement without impairment of balance; (3). Bilateral disease with mild to moderate disability and impaired postural reflexes but physical independence; (4). Severely disabling disease but still able to walk or stand unassisted; (5). Confinement to bed or wheelchair unless aided (32). The MDS-UPDRS-m Part III assesses problems in motor function on a 5-point scale: O (no problems), 1 (minimal), 2 (mild), 3 (moderate), and 4 (severe) (33). Perceived stress was assessed with the Impact of Event Scale (IES) (34); depression, with the Patient Health Questionnaire-9 (PHQ-9) (35, 36); anxiety, with the Generalized Anxiety Disorder Scale-7 (GAD-7) (37); subjective incompetence, with the Subjective Incompetence Scale (SIS) (29, 30); and demoralization with the Demoralization Scale (DS) (38). All scales have adequate reliability and validity, and have been widely used in research, including research on Parkinson's disease. The SIS is a 12-item, 4-point response scale, with Cronbach's alpha for internal consistency in a cancer cohort of 0.90 and concurrent validity established with the denial, disengagement and self-blame subscales of the Brief COPE measure. Examples of items include “Were you puzzled, indecisive and uncertain as to what actions, if any, you should take?” and “Did you feel that you were running out of ideas to handle the situation?” (27, 28). The DS consists of 24 items and comprises four subscales: loss of meaning and purpose (α = 0.88), disheartenment (α = 0.88), dysphoria (α = 0.80), and sense of failure (α = 0.76). The scale has a good overall internal consistency (α = 0.84). Items are rated on a 5-point Likert scale ranging from 0 (never) to 4 (all the time). A total score for demoralization is calculated by summarizing the single subscale scores with higher scores indicating higher levels of demoralization (38).

### Statistical Analysis

A rule to establish the sample size for path analysis is multiplying the number of measurement variables by 10 (39). With 5 variables included in the analysis, a total of 50 subjects would have been acceptable. A larger sample size, however, would be more convincing. In a separate study we found that the prevalence of demoralization among outpatients with Parkinson's disease was 18.1% (25). With this prevalence ratio, to reach a margin error of 8%, at a confidence level of 95%, at least 89 participants were required (40).

Structural equation modeling with path analysis (SEMPA) was conducted using AMOS version 23 (41). The variables studied were demoralization, perceived stress, subjective incompetence, anxiety, and depression. All variables were treated as continuous. The normality of their distributions was determined using the Kolmogorov-Smirnov and the Shapiro-Wilk tests (42–44). Statistical analyses other than SEMPA were performed using SPSS version 23 (45). The statistical significance level was set at *p* ≤ 0.05, and all statistical tests were two-tailed.

The medians and interquartile ranges (IQRs) were used to summarize the data on the variables measured because the distributions were not normal. Their inter-correlations were examined by calculating Spearman's rho correlation coefficients. SEMPA was used to obtain: (1) the direct effects of subjective incompetence, anxiety, depression, and perceived stress to demoralization; and (2) intermediary effects of (a) depression on demoralization through subjective incompetence, (b) anxiety on demoralization through subjective incompetence; and (c) perceived stress on demoralization through anxiety, depression, and subjective incompetence. All residuals of endogenous variables were assumed to be uncorrelated except for the residuals of anxiety and depression, which were assumed to be correlated. To obtain an over-identified model, the coefficient was preset to the partial covariance between anxiety and depression controlled for subjective incompetence.

The model fit was evaluated by a set of fit indices, including the chi-squared (χ^2^) statistic, Tucker Lewis index (TLI), comparative fit index (CFI), and Root Mean Square Error of Approximation (RMSEA). An excellent fit is indicated if the χ^2^ statistic is non-significant with CFI and TLI ≥ 0.95 and RMSEA ≤ 0.05. Given the non-normal distribution in the measured variables, *p* values and confidence intervals were estimated using bias-corrected bootstrapping with 5,000 re-samples to test the significance of proposed direct and intermediary effects.

## Results

There were 95 participants in this study (participation rate = 71.4%). The majority were male (66.3%), white (91.6%), married (70.5%), and had a college degree or higher (70.5%). The age range of the sample was 44–84 years (mean = 67.81 years, SD = 8.39 years). Only 2 subjects (2.1%) were cigarette smokers; 34 (35.8%) reported drinking alcoholic beverages; and none had a history of mental disorder. The average time since Parkinson's disease diagnosis was 2.87 ± 1.12 years, and the participants were mostly in Hoehn and Yahr stages I or II (87.4%). Dyskinesia was noted in 21 (22.1%) and hypertension in 45 (47.8%). All but 4 were treated with antiparkinsonian medications (95.8%, *N* = 91); 60 (84.2%) were treated with L-dopa; and 17 (22.1%) had received deep brain stimulation. There were no dropouts or missing data.

**Figure 1:**
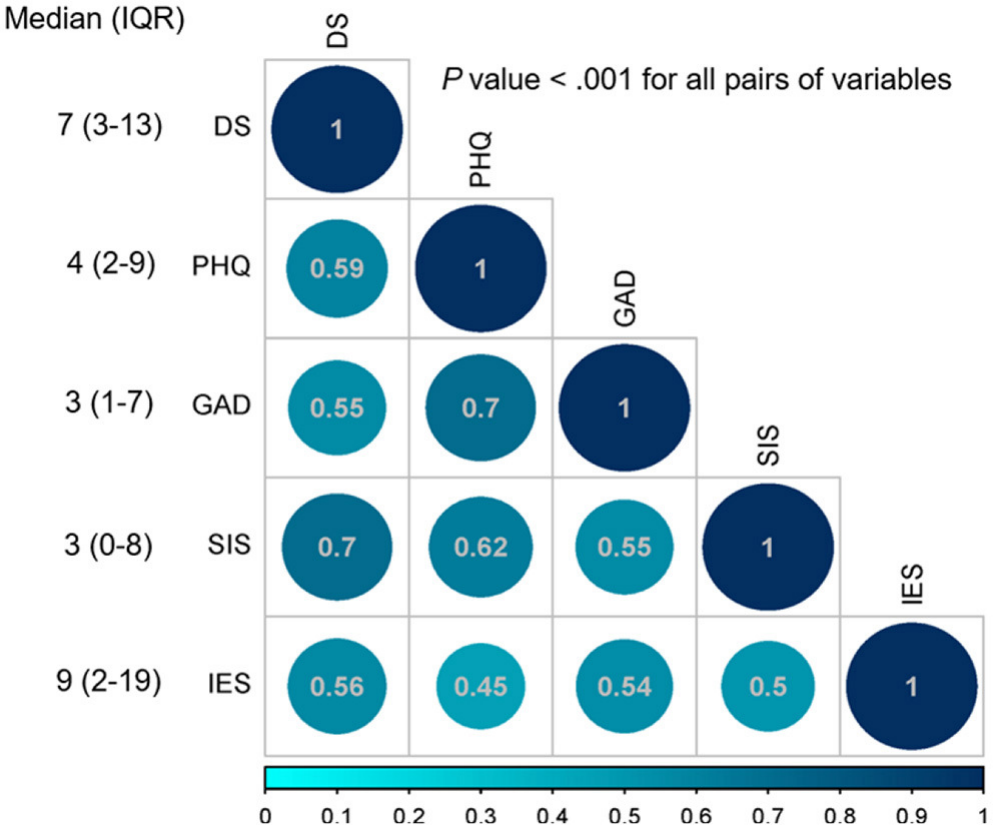
Spearman's rho correlation coefficients, median, and interquartile range (IQR). DS, Demoralization scale; PHQ-9, Patient Health Questionnaire-9; GAD-7: General Anxiety Disorder Scale-7; SIS, Subjective Incompetence Scale; IES, Impact of Events scale.

Intercorrelations of demoralization, subjective incompetence, anxiety, depression, and perceived stress were positive and significant. In particular, demoralization was positively and significantly associated with perceived stress, anxiety, depression, and subjective incompetence. The median DS score was 7 and the IQR was 3 to 13 (Figure 1).

**Figure 2:**
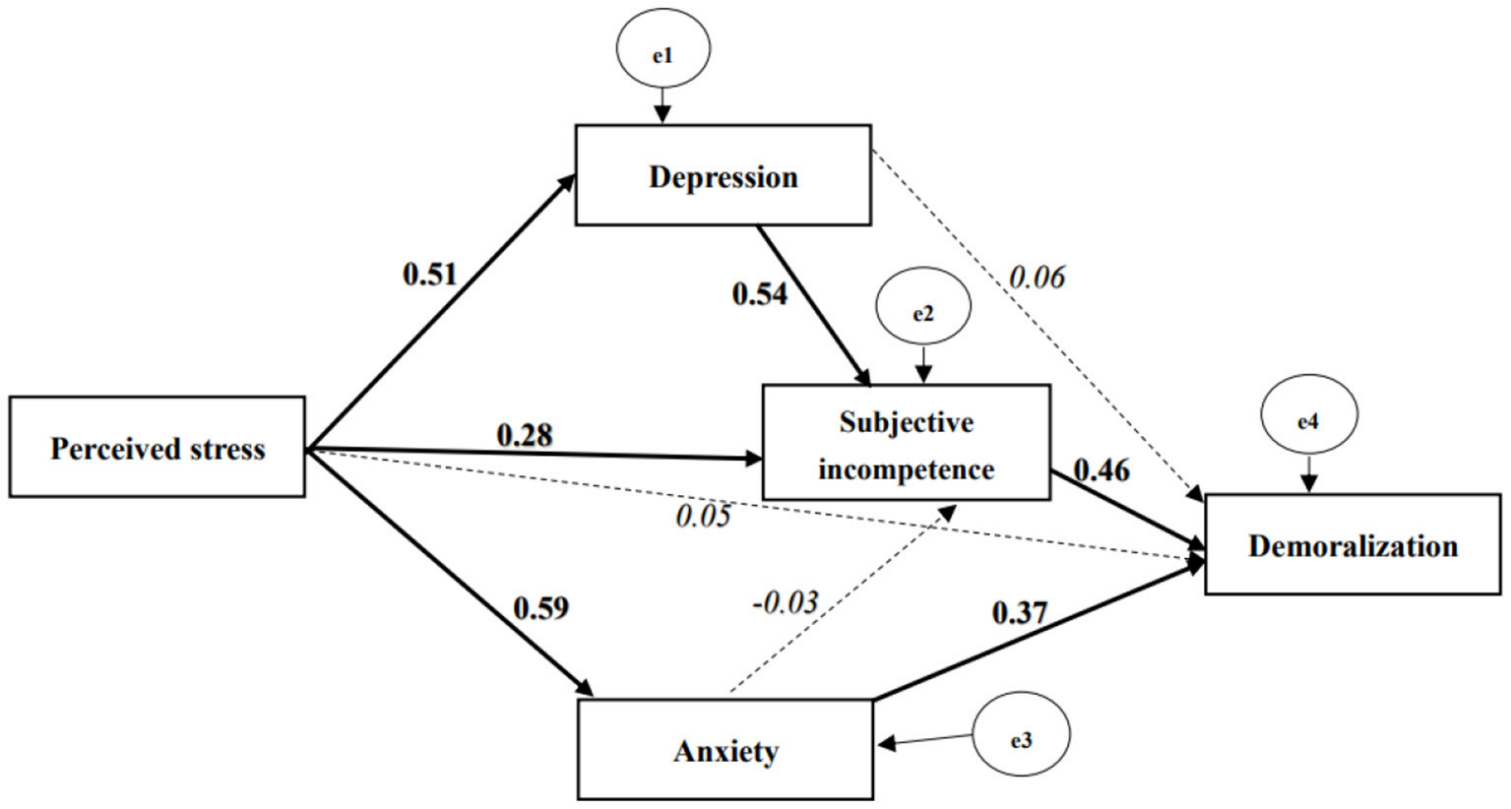
Path model of the proposed effects of perceived stress, subjective incompetence, depression, and anxiety on demoralization with standardized coefficients. Continuous line, bold font indicates significant parameter (*p* value ≤ 0.05); dash line, Italic font indicates non-significant parameter; the e1 and e3 were assumed to be correlated, and the coefficient was fixed to the value of partial co-variance between depression and anxiety controlled for perceived stress. Model fit, Chi-square <0.001, *P* value = 1.000; TLI = 1.04; CFI = 1.00; RMSEA <0.001.

The model fit indices suggested an ideal overall model fit for the data (*p* = 1.000*;* TLI = 1.04; CFI = 1.00; RMSEA <0.001). Both subjective incompetence (β = 1.06, 95% CI: 0.54 to 1.62, *p* < 0.001) and anxiety (β = 1.09, 95% CI: 0.33 to 1.92, *p* = 0.007) had a positive and significant direct effect on demoralization. However, the direct effects of perceived stress (β = 0.05, 95% CI: −0.17 to 0.27, *p* = 0.733) and of depression (β = 0.16, 95% CI: −0.49 to 0.75, *p* = 0.631) on demoralization were non-significant (Figure 2).

The bootstrapping method yielded a positive and significant indirect effect of depression on demoralization through subjective incompetence (β = 0.67, 95% CI: 0.27 to 1.32, *p* < 0.001), whereas a similar indirect effect was not observed from anxiety on demoralization (β = −0.04, 95% CI:−0.54 to 0.46, *p* = 0.882). Furthermore, the total indirect effect of perceived stress on demoralization was found to be significant and positive (β = 0.52, 95% CI: 0.32 to 0.78, *p* < 0.001). This total indirect effect consisted of five specific paths: (a) via subjective incompetence to demoralization (β = 0.14, 95% CI: 0.05 to 0.29, *p* = 0.002); (b) via anxiety to demoralization (β = 0.22, 95% CI: 0.06 to 0.44, *p* = 0.006); (c) via depression to demoralization (β = 0.03, 95% CI: −0.11 to 0.15, *p* = 0.610); (d) via depression to subjective incompetence to demoralization (β = 0.13, 95% CI: 0.05 to 0.32, *p* < 0.001); and (e) via anxiety to subjective incompetence to demoralization (β = −0.01, 95%CI: −0.12 to 0.09, *p* = 0.875). Neither perceived stress nor depression had a significant direct effect on demoralization, thus leading to the conclusion that their relationships to demoralization were fully mediated (Table 1).

**Table 1:** Hypotheses testing for specific indirect effects on demoralization.

**Path**	**Standardized coefficient**	**Unstandardized coefficient**	**SE**	**Bias-corrected 95% CI**		***P* value**
				**Lower**	**Upper**	
Perceived stress: total indirect effect	0.50	0.52	0.12	0.32	0.78	0.000
→ Anxiety → demoralization	0.21	0.22	0.10	0.06	0.44	0.006
→ Depression → demoralization	0.03	0.03	0.06	−0.11	0.15	0.610
→ SI → demoralization	0.13	0.14	0.06	0.05	0.29	0.002
→ Anxiety → SI → demoralization	−0.01	−0.01	0.05	−0.12	0.09	0.875
→ Depression → SI → demoralization	0.13	0.13	0.06	0.05	0.32	0.000
Depression → SI → demoralization	0.25	0.67	0.26	0.27	1.32	0.000
Anxiety → SI → demoralization	−0.01	−0.04	0.25	−0.54	0.46	0.882

*SI, subjective incompetence; SE, standard error for unstandardized coefficient; CI, confidence interval*.

## Discussion

Consecutive outpatients with Parkinson's disease were evaluated for perceived stress, depression, anxiety, subjective incompetence, and demoralization. Path analysis revealed that the path from perceived stress to demoralization (total effect) was statistically significant and completely mediated by subjective incompetence, depression, and anxiety. Among all variables, subjective incompetence was the largest contributor to demoralization. Subjective incompetence fully mediated the path from depression to demoralization but it was not a mediator in the path from anxiety to demoralization. These findings are consistent with the hypotheses that (a) in the differential diagnosis between depression and demoralization, subjective incompetence is the clinical hallmark of demoralization (8) and (b) the occurrence of subjective incompetence converts depression into demoralization (9).

A second, minor path was discovered, independent of subjective incompetence, going from perceived stress to demoralization via anxiety. Some studies indicate that anxiety levels are an important predictor of hopelessness (46). More research is needed to determine if the rise of anxiety sets in motion a rapid progression from subjective incompetence to helplessness, hopelessness, and eventual suicide. Such rapid progression may create an appearance of a direct path from perceived stress to demoralization in this cross-sectional study in which the more advanced stages of the demoralization cascade (helplessness and hopelessness) were not captured by the Subjective Incompetence Scale and were not independently assessed.

The pathophysiology of subjective incompetence and demoralization is incompletely understood. Available evidence suggests that subjective incompetence may be viewed as a manifestation of a “top-down” process triggered by a failure of cortical mood regulation (emotion modulation) (47). Suboptimal performance of executive function may interfere with the goal-directed planning necessary to meet the challenge of the stressful situation created by the progressive neurodegeneration and its catastrophic behavioral and motor results, in turn compounding the perceived stress. Activity and functional connectivity of the medial orbitofrontal cortex has been shown to be related to hope and trait hope (48). An extreme form of demoralization noted in medically ill patients has been called “giving up-given up complex” or “give-up-it is”, and has been attributed to dopamine disequilibrium and resulting dysfunction in the frontal subcortical circuits (49, 50). Depression, on the other hand, may be interpreted as a manifestation of a “bottom-up” process precipitated by a failure of sub-cortical mood regulation (emotion processing) (51). This interpretation is supported by the well-documented role of the dopamine mesolimbic system in depression that explains the anhedonia, anergia, and abulia, often present in major depressive disorder (52, 53), but absent in demoralization.

Several psychotherapeutic approaches have been developed to modify the perception of stress and replace negative cognitive distortions of self and stressful situations with positive, more precise, and more realistic appraisals. Examples are cognitive-behavioral psychotherapy, wellbeing therapy, and meaning-centered psychotherapy (54–56). The efficacy of cognitive-behavioral psychotherapy in Parkinson's disease has been demonstrated for depression and anxiety (57), but it is unclear whether the outcome measures used in these studies also captured demoralization because subjective incompetence was not assessed. As far as we could determine, the efficacies of wellbeing therapy and meaning-centered psychotherapy in patients with Parkinson's disease have not yet been studied. More research is needed on treatment modalities specifically targeted at subjective incompetence. Understanding the mediating role of subjective incompetence between perceived stress and demoralization would pave the way for process-based psychotherapy for demoralization (58). Better understanding of pathophysiology may lead to pharmacological interventions as well.

### Limitations

Participants were outpatients at a single academic hospital, thereby limiting generalizations to patients with mild and moderate disability in similar centers. The study sample consisted mainly of people who were older, white, male, married, and had a college degree. Results may have been different with a more diverse sample. The iatrogenic effects of medications used to treat Parkinson's disease might be unmeasured confounders. Positive scores on the scales employed are not the same as clinician diagnoses. The depression and anxiety scales used in this study (PHQ-9 and GAD-7) correlate only with major depressive disorder and generalized anxiety disorder respectively, and not with other types of depression or anxiety. A specific scale to assess the presence and intensity of suicidal ideation, such as the Scale for Suicide Ideation (SSI), was not used (59–61).

The cross-sectional design precludes causal inferences. Although we used the word “effect” to describe the results because it is the appropriate statistical term, path analysis clarifies the correlations and indicates the strength of the hypothesized causal model but it does not establish causation. In order to establish the direction of causality, an experiment has to be conducted in which participants are randomly assigned to a treatment and a control group. Rather than demonstrating causality, path analysis exposes the logical consequences of a causal model assumed *a priori*.

### Strengths

Participants were evaluated and diagnosed by a movement disorders neurologist (A.P.). A wide range of demographic, clinical, and treatment-related variables was assessed with valid and reliable scales that have previously been widely used in research and have been used in patients with Parkinson's disease. The research is timely, given that (a) demoralized patients with Parkinson's disease are more likely to have suicidal ideation than controls (27), and (b) although studies of suicide in Parkinson's disease led to mixed results in the past, a recent study based on a large register settled this issue by finding that Parkinson's disease is associated with an increased risk of suicide that is not fully explained by higher rates of mental disorders (62).

## Conclusion

Two paths from perceived stress to demoralization were identified, a major one via subjective incompetence and a minor one via anxiety. Our results invite further study of how they differ and their interrelations in a diverse sample including patients with more advanced disease. The contribution of certain variables known to intervene between perceived stress and demoralization and not assessed in this research, such as resilience and perceived social support, should be examined (63, 64). The path from demoralization to suicide should be further investigated.

Clinical trials should identify the most efficacious combination of treatment modalities aimed at reducing demoralization. Early treatment and reversal of subjective incompetence and anxiety in Parkinson's disease could potentially prevent the escalation of demoralization and its adverse impact on health-related quality of life and eventual suicide.

## Data Availability Statement

The raw data supporting the conclusions of this article will be made available by the authors, without undue reservation.

## Ethics Statement

The studies involving human participants were reviewed and approved by Yale University Institutional Review Board. The patients/participants provided their written informed consent to participate in this study.

## Author Contributions

JdF was the primary investigator and author, contributions included developing the hypothesis, reviewing the literature, formulating the research protocol, formulating the statistical analysis, interpreting the findings, and writing and editing the manuscript. BZ contributed with formulating the analyses, interpreting the data output, helping with writing and editing the manuscript, performing the statistical analysis, and making all tables and figures. RK contributed with inputting the data, developing the hypothesis, interpreting the findings, and editing the manuscript. AP contributed with participant recruitment, screening potential participant, performing the UPDRS-m scoring, and editing the manuscript. BK and EL contributed with interpreting the findings and writing and editing the manuscript. All authors contributed to the article and approved the submitted version.

## Conflict of Interest

The authors declare that the research was conducted in the absence of any commercial or financial relationships that could be construed as a potential conflict of interest.

## Publisher's Note

All claims expressed in this article are solely those of the authors and do not necessarily represent those of their affiliated organizations, or those of the publisher, the editors and the reviewers. Any product that may be evaluated in this article, or claim that may be made by its manufacturer, is not guaranteed or endorsed by the publisher.
